# Supplementary figures and images for: The Effect of Cognitive Resource Competition Due to Dual-Tasking on the Irregularity and Control of Postural Movement Components
Source: Entropy (Basel). 2019 Jan 15;21(1):70. doi: 10.3390/e21010070 (PMC7514179; doi:10.3390/e21010070)

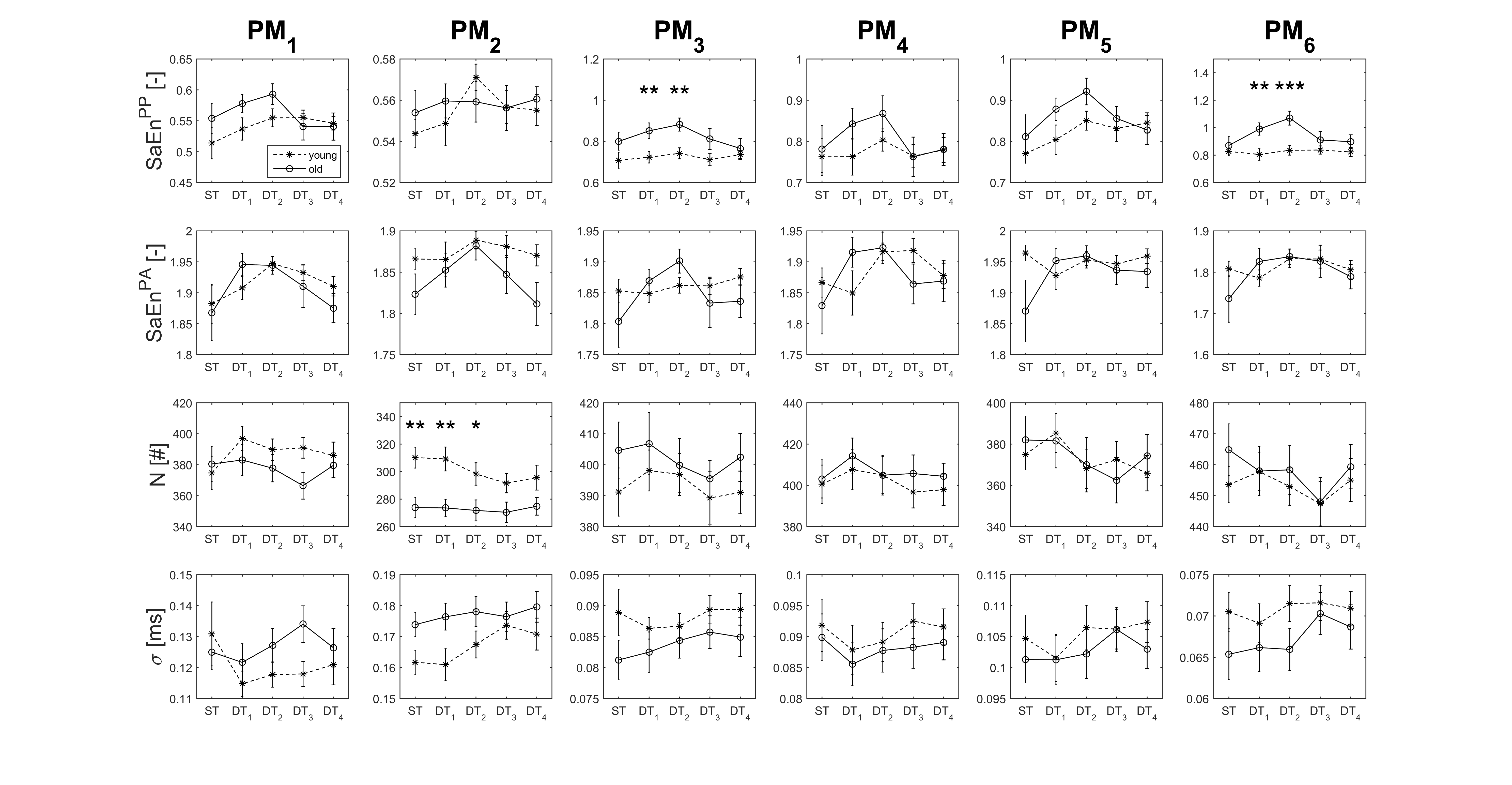

Supplement: Supplementary file 1 [file entropy-21-00070-s001.zip › Age_effects.png]

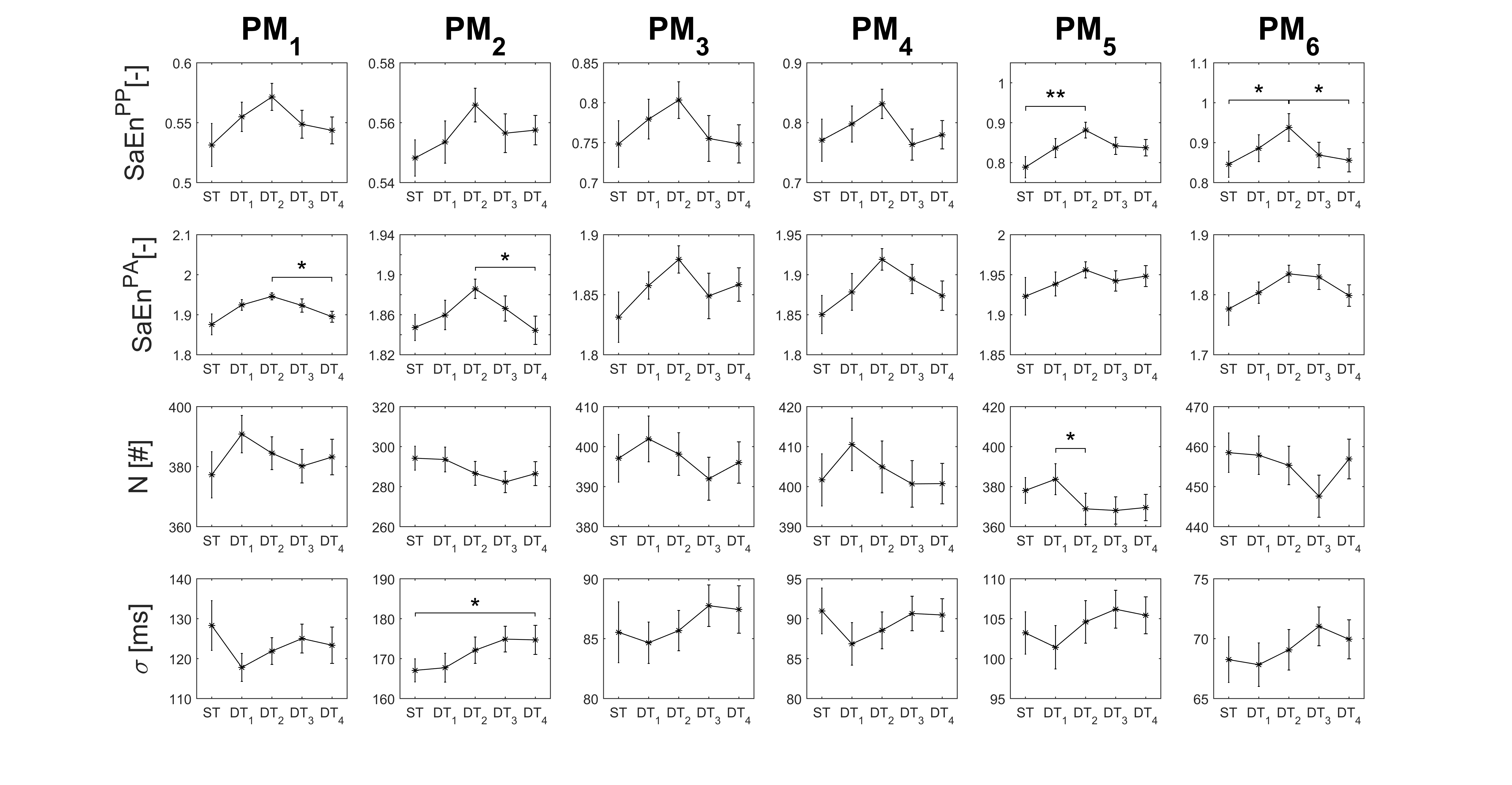

Supplement: Supplementary file 1 [file entropy-21-00070-s001.zip › DualTasking_effects.png]

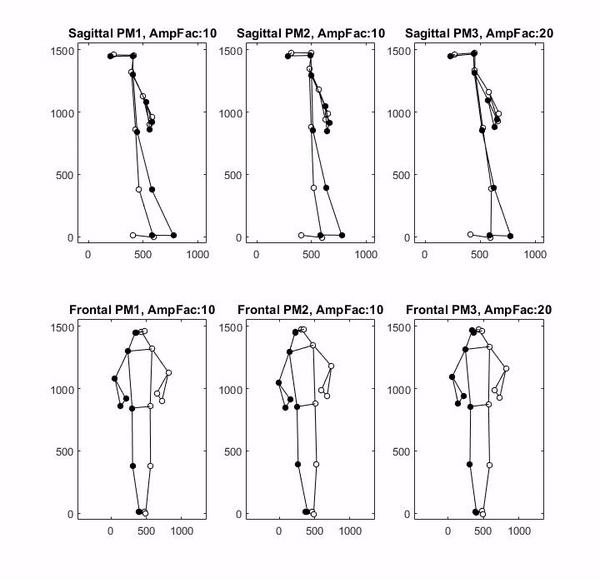

Supplement: Supplementary file 1 [file entropy-21-00070-s001.zip › Visualization_PM1-PM3.gif]

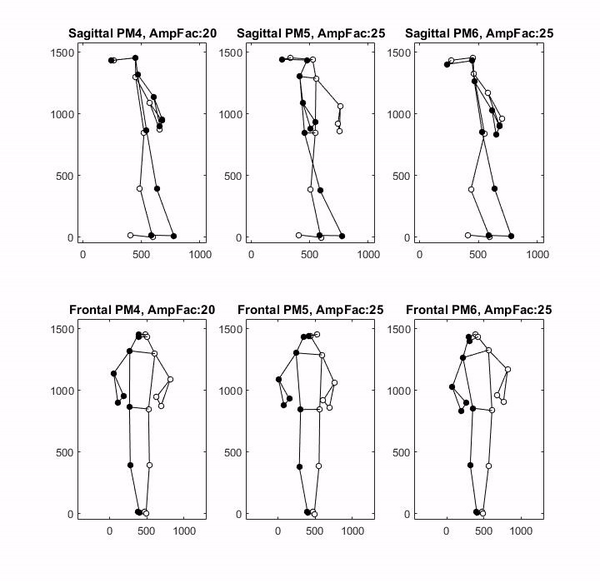

Supplement: Supplementary file 1 [file entropy-21-00070-s001.zip › Visualization_PM4-PM6.gif]
